# Implementation of Family Integrated Care in the Neonatal Intensive Care Unit, University Hospitals Sussex, UK

**DOI:** 10.3390/children13020195

**Published:** 2026-01-30

**Authors:** Natalia Hounsome, Heike Rabe, Eleanor Turk, Pratyush Saha, Farid Ebrahimjee, Ramon Fernandez, Adelina Pellicer, RISEinFAMILY Consortium (European Union, MSCA-RISE-H2020)

**Affiliations:** 1 Department of Global Health and Infection, Brighton and Sussex Medical School, University of Brighton and University of Sussex, Brighton BN1 9PX, UK; 2 Department of Academic Paediatrics, Brighton and Sussex Medical School, University of Brighton and University of Sussex, Brighton BN1 9PX, UK; 3 Department of Neonatology, University Hospitals Sussex NHS Foundation Trust, Brighton BN2 5BE, UK; 4 Department of Neonatology, La Paz University Hospital, 28046 Madrid, Spain; 5 Hospital La Paz Institute for Health Research-IdIPAZ, 28046 Madrid, Spain

**Keywords:** neonatal intensive care unit, family integrated care, economic evaluation, UK

## Abstract

**Highlights:**

**What are the main findings?**

- The duration of stay on ventilation and high-dependency care was lower after implementing FICare;
- The cost of stay in NICU was lower for babies receiving FICare.

**What are the implications of the main findings?**

- This was a pragmatic, non-randomised, non-controlled study based on economic evaluation and clinical audit, which provided evidence on FICare implementation in real-life conditions;
- The study suggests that FICare may be resource-and cost-saving due to a decrease in babies’ stay in high-dependency care.

**Abstract:**

**Background/Objectives:** Family Integrated Care (FICare) is a model of care for preterm or critically ill infants in which families are considered equal partners with clinical teams and are fully integrated into all aspects of care and decision-making. In this study, we conducted a health economics study of FICare implementation in the UK, as part of the EU-funded international, interdisciplinary, and intersectoral project RISEinFamily. **Methods:** An economic evaluation of healthcare services and an audit of clinical outcomes for infants admitted to the Royal Sussex County Hospital and the Princess Royal Hospital neonatal units in 2021 (at the start of FICare) and 2024 (when FICare was fully integrated into clinical practice) were conducted. Anonymized data on hospital admissions were downloaded from the hospital database. Infants with a duration of stay in the NICU of more than 20 days were included in the analysis. The cost of NICU stay was estimated using the Health Resource Group codes. **Results**: The average duration of infants’ stay in NICU was similar before and after the implementation of FICare (47 days (SD 29) in 2021 and 47 days (SD 31) in 2024). However, the infants who received FICare spent fewer days in high-dependency care; on average, 10 days in 2024 and 13 days in 2021. The duration of invasive ventilation fell by 12% and the duration of CPAP by 26% after introducing FICare. The total cost per baby stay in NICU was GBP 63,279 (USD 87,021) in 2021 and GBP 59,284 (USD 75,777) in 2024. **Conclusions:** Although the changes did not reach statistical significance, the study suggests that FICare may be resource- and cost-saving due to reducing infants’ stays in high-dependency care.

## 1. Introduction

Family Integrated Care (FICare) in the NICU is an evidence-based model of care that actively involves and empowers families to participate in the care of their preterm or critically ill infants [1]. Historically, NICUs have operated under a clinician-centred model, in which an infant is viewed as a medical patient in a high-technology environment, with only trained professionals able to provide care [2]. In practical terms, this model manifested in strict visiting hours and medical teams holding complete authority over infant care and providing information to families on a “need-to-know” basis. The primary justification was infection control, securing efficiency in providing life-saving interventions and protecting families from the psychological trauma of seeing their critically ill infants and stressful medical procedures [3,4].

In the 1970s, studies on maternal–infant bonding and skin-to-skin (kangaroo) care [5] laid the foundation for Family-Centred Care, which recognises the importance of family knowledge, values, and beliefs, as well as the need for active family participation in all aspects of care and decision-making [6,7]. In the early 2000s, the Family Integrated Care (FICare) model emerged, grounded in the main principles of Family-Centred Care. Inspired by the Humane Neonatal Care Initiative in Estonia [7], the Canadian team at Mount Sinai Hospital in Toronto developed a structured FICare approach (FICare™), consisting of a parent education curriculum; nurse training to become coaches; psychosocial support for parents, including peer support from “veteran parents”; and physical infrastructure changes in NICU [8]. A large multinational randomised controlled trial conducted in Canada, Australia, and New Zealand (2012–2015) showed increased baby weight and high-frequency exclusive breastmilk feeding over 3 weeks, and decreased parent stress and anxiety at discharge [9].

The Close Collaboration with Parents™ programme in Finland primarily focused on training NICU staff to facilitate culture change and establish better parent–staff collaboration [10]. The program involves mentors guiding staff through bedside practices, focusing on understanding infant cues, integrating parents into all aspects of care, and preparing for hospital discharge. The primary outcomes of this program included improved parental presence, increased skin-to-skin care, and a shorter hospital stay [11].

The Alberta Family Integrated Care model (Alberta FICare™) promoted a holistic approach to NICU care, focusing on education, relational communication, and support of carers to create a family-friendly NICU environment and foster partnership with families [12]. The programme incorporates online educational sessions, support from peer family mentors, engaging families in routine caregiving tasks (e.g., feeding, changing nappies, bathing, and providing comforting touch) and encouraging caregivers to participate in bedside medical rounds, ask questions, share observations, and contribute to the infant care plan. The model enablers included a family-friendly NICU space to better accommodate families (reclining chairs, kitchen facilities, and sleeping options for parents) and partnerships among health care providers, families, and community services. The program was successfully trialled in five Level II NICUs across Alberta, showing a decrease in NICU stays, fewer emergency department visits, fewer hospital readmissions after discharge, and improved maternal psychological health [13].

Mobile-enhanced Family Integrated Care for preterm infants (mFICare) was developed in the USA to increase uptake and outcomes of FICare [14]. A mobile application, We3health™, was developed in partnership with parents and multidisciplinary teams to supplement in-person FICare training and enable caregivers to access training materials at any time or place. The app was also used to record carers’ engagement with the programme, take notes and capture images, provide advice to carers, and collect data through automated surveys. The study found that the mFICare protocol reduced nosocomial infection rates in preterm infants and depression and stress in mothers after NICU discharge [15].

The adaptations of FICare models were introduced in Europe (Spain, the Netherlands, and the UK) [16,17,18]; China [19,20]; the US [21]; Turkey [22]; Uganda [23]; and other countries. A recent meta-analysis of 13 randomised controlled trials with a total sample of 3005 preterm infants confirmed significant improvements in infant outcomes, including breastfeeding rates, weight gain, sleep duration, and a reduction in the one-month readmission rate [24].

While the beneficial effects of FICare were clearly demonstrated, the feasibility of implementing FICare in low-resource settings remains questionable. In 2021, an EU-funded international, interdisciplinary, and intersectoral project, RISEinFamily, started, focusing on the adaptation of FICare in neonatal care units across the world, including high-, low-, and middle-income countries (the Netherlands, Romania, Spain, Turkey, the UK, and Zambia) [25]. As part of this project, we conducted a mixed-methods study to examine the resources and value-for-money of introducing and scaling up the FICare intervention. Whilst the overall study is still ongoing, we present in this article an economic evaluation of FICare delivery at the neonatal units at the Royal Sussex County Hospital and the Princess Royal Hospital in the UK. Our objectives were (i) to characterise resources and costs required to deliver FICare and (ii) to compare the cost of hospital stay at the beginning of introducing FICare in 2021 and when FICare was fully integrated into clinical practice in 2024.

## 2. Methods

### 2.1. Study Design

This was a health economics evaluation study of the implementation of the Family Integrated Care Quality Improvement project at the University Hospitals Sussex, UK. The study included two components: (i) identifying resources and costs associated with delivering FICare in the NICU and (ii) an audit of NICU admissions at the beginning of introducing FICare in 2021 and when FICare was fully integrated into clinical practice in 2024. The economic evaluation was conducted from a healthcare provider’s perspective.

#### 2.1.1. Economic Evaluation of FICare

FICare is a complex intervention that aims to improve the health and quality of life of newborns, families, and hospital staff. The intervention is deeply embedded in clinical practice and has become a part of usual care. Identifying and costing components related to FICare are challenging. An analytical framework was developed in collaboration with clinicians and ward managers to identify resources required to deliver FICare, including staff, facilities, and procedures. We focused on the FICare running costs, since the set-up costs were difficult to estimate retrospectively, given that different elements of this intervention had been developed and piloted within the Kent, Surrey, and Sussex Operational Delivery Network (KSS ODN) since 2019 (before the RISEinFamily project started). Therefore, the one-time upfront costs (such as designing the FICare intervention, developing training materials for families and staff, conducting consultations with stakeholders, piloting different elements of care, collecting families’ feedback, etc.) were outside the scope of this study.

In this article, we focused on the running costs of FICare: training healthcare professionals in FICare, carers’ training, FICare family-led ward rounds, ward environment, and psychological support for families and staff. Data on FICare activities, their frequency and duration, staff involvement, and their salary grades were collected from FICare lead nurses and ward managers.

#### 2.1.2. Clinical Audit

The data on resource use and clinical outcomes were collected through an audit of the implementation of the Family Integrated Care Quality Improvement project for parents whose infants were admitted to neonatal units (audit N 2474). The audit was approved by the Clinical Audit Department of the University Hospitals Sussex NHS Foundation Trust on 4 April 2025. The audit covered admissions to the Royal Sussex County Hospital and the Princess Royal Hospital Neonatal Units in 2021 (at the start of FICare) and 2024 (when FICare was fully embedded into clinical practice). The aim of the audit was to compare the costs and baseline values for the core outcome components of FICare in 2021 with those achieved in 2024. Only infants with a stay duration > 20 days were included, as FICare training takes some time to have an effect.

### 2.2. The Setting

The Trevor Mann Baby Unit (TMBU) at the Royal Sussex County Hospital in Brighton is a level 3 Surgical Neonatal unit providing care for preterm and critically ill newborns. TMBU is linked to the level 1 Special Care Baby Unit (SCBU) at Princess Royal Hospital, which provides care for infants with a gestation age of 32 weeks or more. TMBU is a referral centre for the uplift of neonatal care from the Kent, Surrey, and Sussex (KSS) area, with a population of over 4.5 million. The Unit also provides clinical placements for medical and nursing students from the Universities of Brighton and Sussex. The KSS Operational Delivery Network (KSS ODN) assures governance arrangements for neonatal critical care units within the Southeast Coast area. The level 3 unit (TMBU) comprises three wards, providing three levels of nursing and medical care: Intensive Care, High-Dependency, and Special Care. The total number of beds is 27 (9 in Intensive Care, 8 in High-Dependency, and 10 in Special Care). Bed occupancy rates are 89% or higher. The level 1 unit (SCBU) provides eight cots.

### 2.3. Multidisciplinary Teams

Staff across both hospital sites (TMBU and SCBU) include 12 neonatology consultants, 2 specialist doctors, 21 resident doctors in training, 14 advanced neonatal nurse practitioners, 76 nurses at TMBU, and 10 at SCBU. Staff numbers on a shift vary depending on acuity and capacity, with an average of eight doctors covering the two units and one doctor/advanced neonatal nurse practitioner delivering transitional and postnatal care at the maternity wards at both sites. There are, on average, 13 nurses on shift at TMBU, of which nine are trained in the specialist pathway, with a supernumerary nurse in charge (also qualified as a specialist) and one nursing assistant. In SCBU, there are, on average, 3 nurses per shift, including 1 trained in the specialist pathway.

A dietician is available 5 days/week, specialised in assessing, diagnosing, and managing the nutritional needs (including parenteral and enteral feeding) of preterm and sick infants. The dietician provides support to infants who are not growing as expected or who are not tolerating their current feeding regimens (e.g., due to intolerances or surgical conditions). A dietician advises on formula milk, breast milk fortifiers, vitamins for optimal growth and development, and iron supplements.

A pharmacist is available 7 days/week to assist with optimising medication use and ensuring the safety of neonatal patients, including parenteral nutrition and medication error prevention. They tailor therapy to infants’ needs, ensuring that doses and regimens are appropriate and that monitoring is in place, thereby improving the safety of medication use in neonates.

An occupational therapist is also present (5 days/week) whose primary focus is developmental care, supporting bonding with families and infants, and teaching ways to help infants achieve optimal growth and development while on the units. This includes recognising pain and comfort cues, introducing age-appropriate play and stimulation, promoting kangaroo care, and creating a personalised plan to support infant development.

A physiotherapist is available 4 days/week who provides assessments and support to infants and teaching for their families. Physiotherapists provide care for a wide range of conditions, from respiratory conditions to developmental care and positioning, including kangaroo care, positioning, physical activity, and massage. The physiotherapist is involved in respiratory care, helping to maintain clear airways and clear secretions, and prevent respiratory complications. The physiotherapist also manages orthopaedic conditions, such as positional or structural foot deformities and obstetric brachial plexus palsy.

A speech and language therapist is available five days a week to provide support to infants with non-nutritive sucking/swallowing difficulties, often due to prematurity or medical conditions. They assess sucking and swallowing reflexes to establish safe oral feeding (by breast or bottle), including transitioning from tube feeding to oral feeding. They also assess and manage communication difficulties, supporting early communication and interaction between parents and their infants, and advise on ways to overcome barriers to bonding and attachment, such as those created by medical interventions.

There are two named psychotherapists (five days a week) who support the emotional and psychological well-being of parents and siblings of patients in the unit, addressing the challenges of a sick or preterm baby, with a focus on parent–infant relationships and neurodevelopmental outcomes. They offer conversations which focus on trauma processing, bonding for siblings, and dealing with difficult emotions, and act as a point of contact for families to talk about their experiences.

A counsellor is available five days a week through the maternity services to support families, including siblings, during challenging times, and can signpost to other services when necessary.

### 2.4. Care Provision

#### 2.4.1. Neonatal Intensive Care

The level 3 unit receives admissions from within the KSS network and, if needed, from further afield. The unit provides the highest acuity care for infants from 22 weeks’ gestational age to term infants who require ventilation, inotropic support, or surgery. In agreement with the British Association of Perinatal Medicine (BAPM) Categories of Care [26], this includes infants who receive any form of mechanical respiratory support, umbilical central line catheters, peripheral arterial line, replogle tube, chest drain, silo for gastroschisis, infusion of insulin or prostaglandin, exchange transfusion, or therapeutic hypothermia. The ward accommodates 9 beds, including 1 for emergency admission. The patient space includes one room with nine cots, and individual spaces can be separated by divider screens. The typical nurse-to-patient ratio is 1:1, and the doctor-to-patient ratio is 1:3. The usual shift is 12.5 h for doctors and nurses and 7.5–8 h for allied health professionals.

#### 2.4.2. High-Dependency Care

The High-Dependency Unit supports stable preterm and term infants who receive any form of non-invasive respiratory support, parenteral nutrition, continuous infusion of drugs (except prostaglandin and/or insulin), central venous line, tracheostomy, urethral or suprapubic catheter or trans-anastomotic tube following oesophageal atresia repair, airway/nasal stent or observation of seizures, cystic fibrosis monitoring, or barrier nursing [26]. These infants still require highly skilled nursing care but can be cared for with a lower nurse-to-patient ratio of 1:2/3. The unit includes one room with nine cot spaces.

#### 2.4.3. Special Care

The level 1 units at both hospital sites (TMBU and TMBU) support infants who have either progressed from previous levels of care and are preparing for discharge, or infants who require basic monitoring and interventions. These can include neonates who do not fulfil the criteria for intensive or high-dependency care but require oxygen by nasal cannula, feeding by nasogastric or jejunal tube or gastrostomy, the presence of an IV cannula, care of stoma, blood glucose monitoring, phototherapy, or continuous physiological and special observation of physiological variables at least four hourly [26]. There are 10 specialist care cots at TMBU and 8 at SCBU, with a maximum nurse-to-patient ratio of 1:4. The wards particularly focus on preparing infants for discharge and supporting parents in caring for their infants independently.

#### 2.4.4. Transitional Care

Transitional Care is provided on the Maternity ward by one junior doctor/Advanced Neonatal Nurse Practitioner and overseen by senior clinicians. They assist the perinatal multidisciplinary team, attend newborns in the delivery or operating room (e.g., assess newborns or assist with deliveries of preterm infants), and aid and follow up newborns in maternity wards when a mother is hospitalised.

#### 2.4.5. The Feeding Team

All staff are trained to support families with infant feeding; however, there is also a designated feeding team that includes a specialist feeding nurse and confident feeding advisors. Advisors are qualified neonatal nurses or midwives with a special interest in infant feeding, who have completed a breastfeeding course and serve as breastfeeding champions on their units. They are part of the UNICEF UK Baby Friendly Initiative, which partners with UK public services (maternity, neonatal, health visiting, and universities) to improve infant feeding support and parent–baby relationships [27].

The feeding team assesses and supports oral feeding when neonatal nurses require more support. The donor milk is provided by the Southampton Human Milk Bank [28] run by Southampton Hospital Charity, which supports neonatal units across the south of England.

#### 2.4.6. Neonatal Community Outreach Team

The neonatal outreach team consists of two neonatal nurses who provide continuation of care between the units and home. The team works alongside health visitors to help smooth the transition to discharge. They provide support with feeding and growth, referrals, home oxygen, and monitoring.

#### 2.4.7. Outpatient Consultations

Weekly Outpatient Clinics are provided for previously hospitalised and discharged infants by consultants, specialist doctors, doctors in training, and allied health professionals. Patients are followed up in the community, depending on their developmental and physical needs and in accordance with national standards, up to the age of 2 years. The follow-up is provided to infants < 31 weeks or birth weight < 1500 g and all infants with hypoxic–ischemic encephalopathy, congenital malformation, serious illness after birth, and intrauterine growth restriction below the 2nd centile.

#### 2.4.8. Imaging Facility

The facility offers cranial ultrasound, lung ultrasound, and echocardiogram services, performed at TMBU by trained staff, primarily consultants and specialist doctors. Where required, specialist scans are performed by members of the multidisciplinary teams at the Royal Alexandra Children’s Hospital.

#### 2.4.9. Transport

The neonatal transfer service for Sussex is based at the level 3 unit and has a specialist transport ambulance just for neonatal transfers. They work alongside other transfer teams in Kent and Surrey to cover the neonatal network in this region, and the three teams rotate to provide a 24/7 transport service. They complete time-critical emergency transfers, repatriations, and outpatient appointment transfers. The team comprises a doctor/advanced neonatal nurse practitioner and a senior nurse experienced in neonatal transport, and is supervised by the consultant on call for the level 3 unit transfer.

#### 2.4.10. Family Rooms

An optional overnight stay in the family room is offered to the families before infants are discharged from the hospital. The facility includes two en-suite rooms where parents can stay a few nights with their infants, with staff overseeing care and providing advice. The facility also includes a kitchen, a shared shower room, and a milk-expressing room, which is also used for caregiver peer support. There is a fridge and cooking facilities in the kitchen. Sandwiches, fruit, and beverages are supplied by the Early Birth Association charity.

#### 2.4.11. The Ronald McDonald House

The charity-run accommodation includes 18 en-suite rooms available to families with seriously ill children from TMBU and Royal Alexandra Children’s Hospital. This enables parents to stay close to their infants and facilitate sibling stays. There are 10 bedrooms in the hospital building and 8 rooms in the house near the hospital. There is a kitchen space for cooking and laundry facilities. Caregivers can use daycare passes, available for a refundable fee of GBP 25, to spend time away from the ward.

### 2.5. FICare Model of Care

FICare was introduced within the KSS ODN in 2019 and included in the BAPM Family Integrated Care Framework [29]. The key components of FICare include partnership with families, empowerment of families, well-being in neonatal units, culture in neonatal units, and environment in neonatal units.

#### 2.5.1. Partnership with Families

Families are considered equal partners of the multidisciplinary teams and are fully integrated in all aspects of care and decision-making. Families are supported to actively participate in ward rounds, providing feedback and planning infants’ care while on the unit and after discharge. Families’ experiences and feedback inform improvements in care. FICare activities are guided and overseen by the Local FICare Steering Groups, which include families, parent advisory groups, and members of the multidisciplinary team.

#### 2.5.2. Empowerment of Families

Families are provided with education, training, and support to be confident caregivers, fully engage in their infants’ care, and advocate for their needs. This includes individual cot-side training, family classes, access to information outlining parents’ roles as caregivers, and information on support available to families from the hospital, local charities, and third-sector organisations.

#### 2.5.3. Wellbeing on Neonatal Units

The well-being aspects include families’ mental health and well-being, focusing on specialised psychological care, peer support, and education, as well as staff well-being, with a focus on creating a positive workspace to prevent burnout and enhance collaborative teamwork. Families and staff have access to specialist psychological and mental health support, peer-to-peer support, group activities (e.g., yoga, crafting, meditation), local charities, third-sector organisations, and community groups providing well-being support for families and staff. Families also have access to translation services and written information to mitigate language barriers. The FICare model ensures the consistency of care when transferring between units.

#### 2.5.4. Culture on the Neonatal Units

The culture aspect promotes practices that integrate families into the delivery of care. This includes empowering staff to lead the implementation of FICare, providing education and training activities on the philosophy and benefits of FICare, and developing communication, coaching, and mentoring skills. Engagement with families in all aspects of the development and delivery of neonatal care is an essential component of the FICare culture.

#### 2.5.5. Environment on the Neonatal Units

Neonatal units provide family-friendly physical and social environments to minimise family separation and enhance the care experience. This includes a welcoming atmosphere, 24 h open access for families, cot-side reclining chairs, screens for privacy, breast pumps, a family restroom with kitchen facilities, personal storage spaces, a milk-expressing room, and signposting of financial support for families (e.g., for travel costs, food, and parking). Neonatal units should provide an environment and procedures to ensure the consistent transition of care when infants are discharged or transferred to other units.

### 2.6. FICare Delivery

Within KSS ODN, the FICare Network Care Coordinators work with individual units to develop, implement, and track unit-specific changes that support FICare, focusing on learning points and outcomes. NHS England South East has launched a Family Integrated Care website that provides helpful tools and resources to support the FICare model [30]. The resources include a sample FICare Information sheet, an information handout and poster, and information on staff education and support, parent education, the NICU environment, and psychosocial support. The educational materials were co-designed with parents from the advisory groups.

#### 2.6.1. Staff Education

The staff education programme aims to establish effective communication between families and healthcare professionals, build trusting relationships, and involve parents in their baby’s care. The staff learn about the psychological implications of having a preterm or sick baby, the process of involvement of parents in their baby’s life, shared decision making, and resources to support the parents in the NICU. They learn about family-centred medical rounds and self-care approaches to reduce burnout and compassion fatigue. Staff education resources include workshops, refresher training, and online resources. The workshops for nurses are held three times a year in four groups and include a one-day training session. The teaching sessions for doctors are conducted twice a year and last approximately 30 min. A weekly bulletin and WhatsApp groups are used for any FICare updates. A monthly survey is produced to summarise key outcomes and challenges of FICare.

#### 2.6.2. Caregiver Education

Parent education aims to explain the FICare model and infant neurobehaviour, as well as stress and pain, and to involve parents in day-to-day baby care. It includes online self-learning, cot-side training, and peer support. Training workshops for families were piloted but did not receive broad support, as families preferred individual learning for its time flexibility. Online self-learning was identified as the preferred training method in the 2023 caregiver survey. Self-learning provides flexibility and better fits into the daily family routine. Caregivers decide on topics they want to learn and tasks they want to do (e.g., changing diapers, bathing, and skincare). More advanced levels of care may include monitoring fluid intake, skin status, vital signs, non-invasive respiratory support, nasogastric or orogastric feeding, urinary catheter care, and ostomy care. Caregivers record their training goals and wishes on the laminated log sheet kept by the cot.

#### 2.6.3. Cot-Side Training

Cot-side training is conducted by neonatal nurses daily, depending on the infants’ clinical status and caregivers’ availability and willingness. There are no minimum training hours or hours when caregivers must be present on the ward. Nurses provide cot-side training according to caregivers’ goals and wishes. Caregivers record their training in the log sheet, indicating the tasks they can do (“prepare feeds”, “tube feed”, “give medicine”, “perform stoma care”, etc.) and the level of involvement (“with support” or “independently”). A nurse on shift observes caregivers as they perform the tasks and provides additional training/advice if required. A nurse keeps records of caregiver training and the tasks they can do in the Electronic Patient Record System.

#### 2.6.4. FICare Meetings

The level 3 unit runs one-hour coffee mornings once a week for caregivers and health professionals, during which caregivers can ask questions and share their experiences with staff and peers. Coffee mornings are attended by all available staff and caregivers. The level 1 unit is supported by the local charity, the Early Birth Association, which runs monthly coffee mornings for parents.

#### 2.6.5. FICare Family-Led Ward Rounds

The FICare ward rounds are conducted cot-side once a week and last 20–30 min. FICare rounds are attended by nurses, doctors, allied health professionals, and caregivers. This is an opportunity to discuss the baby’s medical condition with caregivers, allowing them to share their views and observations. Before FICare rounds, caregivers are encouraged to complete the Family-led Ward Round Prompt Sheet, which includes personal baby information and issues to discuss at the FICare round (feeding and nutrition, breathing, heart and circulation, brain and development, medication, baby handling, and family visits). There are sections on how the baby managed last week’s goals, a plan for the next days/weeks, caregivers’ notes and observations, and specific questions to the multidisciplinary team.

#### 2.6.6. Psychological Support to Parents

Admission to the NICU is very stressful for parents and involves anxiety, uncertainty, and fear. Psychological support helps parents understand the care process and facilitates families’ adaptation to the demands of their new situation. Psychological support is provided by hospital-based psychologists and counsellors.

#### 2.6.7. Psychological Support to Staff

The trust has a staff support and wellbeing programme which includes free access to counsellors. In addition, the neonatal staff have access to an external confidential counselling provider who runs weekly bookable one-to-one sessions.

#### 2.6.8. Environment

On admission, caregivers of infants receive the Welcome Information Pack, which includes a link to FICare online resources and a QR code for quick access. Caregivers interested in participating in FICare contact clinical staff for further information and guidance, access to training resources, and arranging cot-side training. There is 24 h access for all caregivers to newborns, chairs for skin-to-skin (kangaroo) care, privacy screens, a milk expression room, milk pumps, access to the family area on the ward with a kitchen and shower, lockers for personal belongings, hangers for clothing, the information area with brochures about the hospital, the neonatal unit, FICare, and available support. An overnight stay for families who want to stay close to their infants to provide care can be arranged in the Ronald McDonald House (see Methods Section).

### 2.7. Data Analysis

Anonymised data on hospital admissions were downloaded from the Badger Neonatal Database, which contains maternity, pregnancy, and neonatal care records of infants admitted to neonatal units. This is a real-time database that merges primary care and hospital records. Data on the management of infants in neonatal units are captured in the Standardised Electronic Database (SEND). These data include birth data (location, day, gestation age, sex, foetus number, weight at birth, head circumference, etc.); admission data (date and time, diagnosis, weight, head circumference, temperature, feeding, etc.); data on stay in the neonatal unit (days on different levels of care, ventilation, oxygen, medication, investigations, feeding, breast milk, etc.); and data on discharge (date and time, discharge destination, diagnosis at discharge, weight, head circumference, feeding, breast milk, oxygen at discharge, etc.). Anonymised data for the periods 1 January 2021–31 December 2021 and 1 January 2024–31 December 2024 were downloaded into Excel version 16.0, formatted, and analysed using descriptive statistics. The Neonatal Database records each admission as a separate case. Therefore, recordings for patients with multiple admissions within the same care episode (hospital provider spell) were combined using the patient identification numbers to enable individual-level analysis. We used classification by the British Association of Perinatal Medicine [26] to characterise infants’ stays across different levels of care (intensive care, high-dependency care, special care, and transitional care). The days of stay on different levels of care were costed using Health Resource Group (HRG) costs (clinically similar patient activity groups used within the NHS for the reimbursement of healthcare services and for standardising reporting). We used HRG tariffs from the National Schedule of NHS Costs 2001 and 2023 (the latest costs available at the time of analysis) [31]. The Schedule included tariffs for NHS trusts and NHS foundation trusts with Critical Care service codes CCU13 (Neonatal Intensive Care Unit). The currency codes were as follows: XA01Z (Neonatal Critical Care, Intensive Care); XA02Z (Neonatal Critical Care, High-Dependency Care); XA03Z (Neonatal Critical Care, Special Care, without External Carer); XA04Z (Neonatal Critical Care, Special Care, with External Carer); and XA05Z (Neonatal Critical Care, Normal Care) [31]. To alleviate the effects of inflation, which fluctuated between 0.8% in 2021, 9.6% in 2022, and 2.6% in 2024 [32], we used the average costs for 2001 and 2023 (the latest available costs at the time of analysis).

The HRG costs for patients with multiple admissions within the same hospital provider spell were summed. We also analysed the data on the duration of ventilation and oxygen supply, baby weight at admission and discharge, and the method of feeding at discharge. Student’s *t*-test was used for univariate analysis, and a MANOVA test was applied for multivariate analysis. Due to the skewed nature of cost and resource use data, statistical significance was assessed using the Mann–Whitney U test. The total cost of infants’ stay in the NICU was adjusted for covariates using a generalised linear regression model with a gamma distribution and a log link. Covariates included in the model were gestational age, sex, birth weight, and number of foetuses (considered confounding factors for the duration of hospital stay). The cost difference between 2021 and 2024 was estimated using a nonparametric bootstrap (5000 estimates). Statistical analysis was conducted using STATA 16.1. A *p*-value ≤ 0.05 was considered statistically significant. Costs in pounds sterling were converted to US dollars using the historic annual exchange rate for 2021 and 2024 [33].

## 3. Results

### 3.1. Characteristics of the 2021 and 2024 Baby Cohorts

The average gestational age, birth weight, and discharge weight were not statistically different in the 2021 and 2024 cohorts. The mean gestational age was 30.5 (SD 3.8) weeks in 2021 and 31.2 (SD 4.2) weeks in 2024, and the mean birth weight was 1559 (SD 819) grams in 2021 and 1666 (SD 869) grams in 2024. There was a higher proportion of twins and triplets in 2024 compared to 2021. The proportion of extremely preterm infants and those with extremely low birth weight, intrauterine growth restriction, respiratory distress syndrome, and suspected sepsis was higher in the 2021 cohort; however, these differences were not statistically significant. On discharge, the percentage of infants receiving nasogastric tube nutrition was higher in 2021, while the proportion of breast-fed and bottle-fed infants was higher in 2024 compared to 2021. The overall average hospital stays (47.3 and 47.5 days) and the discharge weights (2327 (SD 867) grams and 2321 (SD 802) grams) were very similar between the 2021 and 2024 cohorts, respectively. The complete characteristics of baby cohorts are presented in the Supplementary Materials.

### 3.2. Resources and Costs of FICare Delivery

Table 1 shows the key components of FICare included and excluded from costing. Historically, many of the FICare elements were a part of routine care. These include, for example, kitchen and shower facilities on the ward, chairs for kangaroo care, privacy screens, milk pumps, and lockers for personal belongings, as well as family accommodation and access to psychological support for families and staff. The above components were not included in FICare costing, as we focused only on the add-on components: printed information about FICare, staff training sessions, cot-side training for families, family-led ward rounds, FICare weekly meetings with peers and staff, and food for carers who stay on the ward.

**Figure 1:**
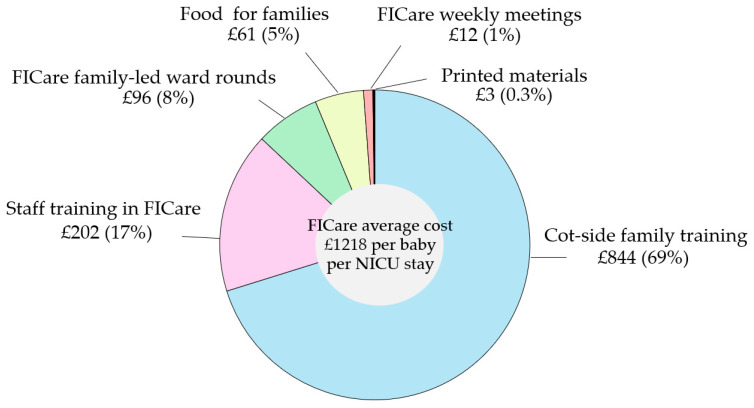
FICare resources and costs. Note that the percentages do not add up to 100 due to rounding.

Figure 1 shows the proportional distribution of FICare resources and costs, with almost 70% allocated to cot-side family training, followed by staff training, ward rounds, family food, and other expenses. The average cost of FICare per baby over their stay in NICU was GBP 1218. This was a conservative estimate since we included in the calculation only infants with a duration of stay > 20 days.

Table 2 shows the use of NICU resources in 2021 and 2024. During the NICU stay, infants spent approximately one quarter (25%) of their time in the Intensive Care ward, a figure similar for both the 2021 and 2024 cohorts. Infants from the 2021 cohort spent, on average, 13 days on the High-Dependency ward and 22 days on the Special Care ward, compared to the 2024 cohort, who spent 10 days on the High-Dependency ward and 25 days on the Special Care ward. The total duration of NICU stay was similar (47 days) for both cohorts.

The 2024 cohort spent, on average, fewer days on ventilation, CPAP, and oxygen support than the 2021 cohort (Table 2). Consequently, the average cost of stay in High-Dependency and Special Care was higher in 2021 than in 2024, although these differences did not reach statistical significance. The total cost of NICU stay was GBP 63,279 (SD GBP 48,195) in 2021 and GBP 59,284 (SD GBP 53,766) in 2024. To adjust for the baseline differences between the 2021 and 2024 cohorts, we conducted an analysis using a generalised linear model. The cost of NICU stay was adjusted using covariates, including gestational age, birth weight, sex, and number of foetuses. The adjusted cost of stay was GBP 61,434 (SD GBP 28,372) in 2021 and GBP 58,213 (SD GBP 30,933) in 2024. The bootstrapped difference in adjusted costs between 2021 and 2024 was GBP 3418 (95% CI: GBP 3625; 10,068). However, this difference was not statistically significant due to wide confidence intervals.

## 4. Discussion

The implementation of FICare is structured around a core framework of “Four Pillars” (staff education and support, parent education, NICU environment, and psychosocial support) and a comprehensive Toolkit designed to guide a cultural shift within NICUs [8]. It involves a fundamental change in NICU culture, flexibility and adaptation, veteran parent engagement, and multidisciplinary commitment. However, the successful implementation of FICare across different countries and settings requires model adaptation, meaning NICU resources should be tailored to the specific needs, size, and constraints of individual units, countries, and socioeconomic contexts. This implies a good understanding of resource allocation in the NICU, which is often overlooked in studies of FICare implementation.

This study was primarily focused on characterising the resources and costs required to deliver FICare in the UK healthcare system. The vast majority of UK hospitals are part of the NHS, which is funded via taxation and provides most services free at the point of use. Therefore, this study was conducted from the NHS perspective, focusing on NICU structure, facilities, staff, services delivered by different departments, and resources required to introduce FICare into clinical practice. In addition to direct FICare costs, we examined whether introducing FICare affected the duration and cost of hospital stay. Our study found that the average cost of FICare per baby was lower in 2024 when FICare was fully integrated into clinical practice. Although the difference in hospital stay between 2021 and 2024 was statistically significant, further adjustment for covariates (gestational age, birth weight, sex, and number of foetuses) yielded a *p*-value of 0.116, indicating that some cohort characteristics may have contributed to the difference in costs. A nonparametric bootstrap of adjusted costs suggests a cost saving of GBP 3418 per baby in 2024; however, the 95% confidence intervals indicate substantial uncertainty around this estimate. This could be due to the high heterogeneity of the infants’ sample, which included preterm and term infants with a wide range of conditions, from congenital diseases to sepsis. An increase in sample size could potentially yield a more definitive answer about the cost-effectiveness of FICare, but this would require including more hospitals or extending the study time horizon. Both approaches have shortcomings, including differences in FICare implementation across hospitals and changes in newborn care practices over time.

It remains debatable whether the outcomes of this study were affected by other factors, such as the COVID-19 pandemic. The latter could potentially bias the results in opposite directions: towards a higher incidence of extreme prematurity on one side, and towards a shorter NICU stay to minimise the risk of COVID-19 infection on the other. It should be noted that there were no confirmed COVID-19 cases in the 2021 study sample, and there was no difference in the duration of NICU stay between 2021 and 2024.

There is a limited number of economic studies of FICare implementation. A cluster randomised controlled trial conducted in Neonatal Intensive Care Units in Canada estimated the cost of infants’ stay in NICU, which was lower in the FICare group USD 39,649 (SD USD 19,741) compared to standard care USD 42,195 (SD USD 20,955), although this difference was not statistically significant [34]. It should be noted that in our study, the overall cost of the NICU stay, GBP 63,279 (USD 87,021), was much higher than in a Canadian study. In addition to differences in health care provision in the level II NICU, this can be explained by different methodological approaches to derive costs: top-down costing in the above study versus bottom-up costing in our study. Top-down costing begins with an overall cost and breaks it down into individual costs, while bottom-up costing estimates individual costs and adds them to arrive at the total cost. The bottom-up approach is considered more accurate but time-consuming.

In addition to the healthcare provider’s perspective, a family perspective on FICare costs should be considered when conducting an economic evaluation. A study of out-of-pocket expenses demonstrated that participating in FICare was not associated with a significant increase in family expenditure (median expenditure of CAD 692 in the FICare group and CAD 773 in the standard care group) [35].

In line with earlier FICare studies, our study demonstrated beneficial effects on infants’ feeding, including increased breast and bottle feeding and decreased use of nasogastric tubes at discharge (Supplementary Materials). Earlier studies reported increases in breastfeeding rates [9,20,36] and a reduction in nasogastric tube retention time [37]. A pre-post FICare study conducted in China reported a decrease in respiratory support time [20], which agrees with our findings that infants receiving FICare spent fewer days on ventilation, CPAP, and oxygen support (Supplementary Materials).

Our study has the following limitations:

1. The analysis was based on a relatively small sample size. Including more hospitals within the KSS ODN may be necessary to address uncertainty about costs and outcomes of FICare.
2. The analysis included infants who stayed in the NICU for > 20 days, which means it did not capture all infants who benefited from the FICare intervention. Different time cut-offs should be investigated.
3. The study focused on short-term FICare outcomes and costs. It did not capture long-term healthcare costs (e.g., post-discharge hospital admissions, unplanned clinic visits, and A&E attendances). Previous studies suggested reduced readmissions and emergency service attendance among infants who participated in the FICare programme [13,36,37].
4. The analysis was based on routinely collected patient data, which does not capture all economic costs associated with FICare (e.g., nurses’ burnout and staff turnover).

In conclusion, our findings suggest that implementing FICare can reduce resource use, particularly high-dependency beds, ventilation, CPAP, and oxygen support. The infants receiving FICare can transition faster from High-Dependency to Special Care, which can free resources for more critically ill newborns. Although we were unable to demonstrate statistically significant differences in costs before and after introducing FICare, our data suggest that FICare delivery is not associated with increased resource use or costs, and, given its well-documented health benefits, it has the potential to be cost-effective.

## Figures and Tables

**Table 1:** Components of FICare included in costing.

FICare Components	Description	Included/Excluded from FICare Costing
Ward environment	24 h access for all caregivers to newborns, chairs for skin-to-skin (kangaroo) care, privacy screens, milk expression room, milk pumps, access to the family area on the ward with kitchen and shower, lockers for personal belongings, hangers for clothing, information area with brochures about NICU, FICare, and available support.	Included: printed information about FICare. Other facilities are available as standard care.
Family training	Self-learning: FICare website, FICare Information sheet, Information Handout and Posters, online education materials, printed information on NICU environment, and psychosocial support. Cot-side training: baby positioning, changing nappies, bathing, skincare, and more advanced care such as monitoring fluid intake, skin status, vital signs, non-invasive respiratory support, naso/orogastric feeding, urinary catheter care, and ostomy care. Peer support: weekly meetings (coffee mornings) with peers and staff.	Included: cot-side training, coffee mornings (staff costs), and printed information. Not included: online training materials.
FICare family-led medical rounds	Once-weekly joint family-led ward rounds to discuss with staff infant care issues, feeding and nutrition, breathing, heart and circulation, brain and development, medication, and family visits	Included: staff costs (nurses, doctors, physiotherapist, occupational therapist, speech and language therapist, dietician/breastfeeding nurse)
Psychological support	Access to hospital-based psychologists and counsellors	Not included in FICare costing: provided as standard care.
Family accommodation	Ten bedrooms in the hospital building and eight rooms in a house located close to the hospital with a kitchen and laundry facilities. Rooming facilities on the ward for an overnight stay before discharge	Not included in FICare costing: available to families across the hospital and to families preparing for discharge from NICU
Staff training	Teaching sessions and workshops, refresher training, and online resources	Included: trainers’ time and staff time attending training sessions
Staff support	Support and Wellbeing Programme for staff, free access to hospital counsellors, and external confidential one-to-one counselling	Not included in FICare costing: available to all staff in the hospital

**Table 2:** Resources and costs at the start of FICare (2021) and when FICare was fully integrated into clinical practice (2024).

Resources and Costs	2021		2024		Significance *
	Mean (SD)	Median (IQR)	Mean (SD)	Median (IQR)	
Ventilation days	3.2 (9.7)	0 (2)	2.8 (8.6)	0 (1)	0.332
CPAP days	14.9 (24.1)	4 (18)	11.1 (17.8)	3 (11	0.155
Oxygen days	19.6 (30.6)	5 (22)	17.2 (26.4)	4 (21)	0.915
Intensive Care days	12.0 (18.0)	5 (11)	12.3 (23.2)	4 (11)	0.156
High-Dependency Care days	12.8 (18.7)	7 (10)	10.1 (11.4)	6 (11)	0.444
Special Care days	21.9 (13.6)	22 (21)	25.1 (16.0)	24 (20)	0.160
Cost: Intensive Care	GBP 23,712 (GBP 35,441)	GBP 7874 (GBP 33,465	GBP 24,181 (GBP 45,719)	GBP 3937 (GBP 33,465)	0.158
Cost: High-Dependency Care	GBP 17,380 (GBP 25,415)	GBP 9538 (GBP 12,263)	GBP 13,836 (GBP 15,603)	GBP 8175 (GBP 16,350)	0.639
Cost: Special Care	GBP 21,433 (GBP 13,595)	GBP 20,843 (GBP 20,843)	GBP 21,005 (GBP 13,593)	GBP 18,711 (GBP 16,160)	0.329
Total cost per patient	GBP 63,279 (GBP 48,195)	GBP 45,420 (GBP 38,281)	GBP 59,284 (GBP 53,766)	GBP 37,934 (GBP 45,451)	0.022

* Mann–Whitney U test was used for skewed data.

## Data Availability

The data presented in this study are available on request from the corresponding author.

## Supplementary Materials

The following supporting information can be downloaded at https://www.mdpi.com/article/10.3390/children13020195/s1. Comparison of infant characteristics at the start of FICare (2021) and when FICare was fully integrated into clinical practice (2024).

## Abbreviations

The following abbreviations are used in this manuscript:

BAPM	British Association of Perinatal Medicine Categories of Care
CPAP	Continuous positive airway pressure
FICare	Family Integrated Care
KSS ODN	Kent, Surrey, and Sussex Operational Delivery Network
NHS	National Health Service
NICU	Neonatal Intensive Care Unit
SCBU	Special Care Baby Unit
TMBU	Trevor Mann Baby Unit
UHS	University Hospitals Sussex
