# Supplementary material for: Implementation of Family Integrated Care in the Neonatal Intensive Care Unit, University Hospitals Sussex, UK
Source: Children (Basel). 2026 Jan 30;13(2):195. doi: 10.3390/children13020195 (PMC12939991; doi:10.3390/children13020195)
Supplement: Supplementary file 1 [file children-13-00195-s001.zip › children-4045252-supplementary.pdf]

children

MDPI

Comparison of infant characteristics at the start of FICare (2021) and when FICare was fully integrated into clinical practice (2024)

Newborn characteristics	2021 (n=131)	2024 (n=155)	Significance test*
Gestation weeks, mean (SD)	30.5 (3.8)	31.2 (4.2)	0.185
Number of fetuses, n (%)			0.643
1	117 (89%)	116 (75%)	
2	14 (11%)	36 (23%)	
3		3 (2%)	
Sex, n (%)			0.587
Female	60 (46%)	76 (49%)	
Male	71 (54%)	79 (51%)	
Birth weight, grams, mean (SD)	1559 (819)	1666 (869)	0.288
APGAR scores, mean (SD)			0.296
1 minute	6.42 (2.42)	6.65 (2.43)	
5 minutes	8.23 (1.66)	8.15 (1.94)	
Main diagnoses, n (%)**			0.235
Prematurity (32-36 weeks)	38 (29%)	49 (32%)	
Prematurity (28-31 weeks)	51 (39%)	51 (33%)	
Extreme prematurity (<28 weeks)	34 (26%)	26 (17%)	
Extremely low birth weight (<1000g)	11 (8%)	5 (3%)	
Intrauterine growth restriction (IUGR)	27 (21%)	18 (12%)	
Respiratory distress syndrome	76 (58%)	78 (50%)	
Sepsis suspected	87 (66%)	88 (57%)	
Neonatal hypoglycaemia	24 (18%)	23 (15%)	
Jaundice	46 (35%)	52 (34%)	
Congenital disorders	10 (8%)	11 (7%)	
Surgery	4 (3%)	1 (1%)	
Weight at discharge, mean (SD)	2327 (867)	2323 (802)	0.797
Feeding at discharge, n (%)			0.311
Bottle	79 (60%)	101 (65%)	
Suckling at the breast	40 (31%)	55 (35%)	
Nasogastric tube	41 (31%)	34 (22%)	
Gastrostomy	2 (2%)	0	
Orogastric tube	1 (1%)	1 (1%)	
Discharge oxygen, n (%)			0.808
yes	28 (21%)	31 (20%)	
no	100 (76%)	103 (66%)	
Duration of hospital stay, days, mean (SD)	47.3 (28.9)	47.5 (31.1)	0.964

*Student's t-test was used for univariate analysis, and MANOVA test for multivariate analysis. Note that percentages do not sum to 100% due to multiple diagnoses.
